# Development and internal validation of an artificial intelligence-driven precision rehabilitation model for ischemic stroke: a retrospective cohort study

**DOI:** 10.3389/fmed.2026.1939735

**Published:** 2026-09-09

**Authors:** Hongbo Zhang, Hanhui Ye, Zongyao Ai, Zhouyue Bai, Qiaoyi Bao, Yiting Wang, Xiaohan Jiang, Xiulin Jiang, Feilong Lu

**Affiliations:** 1 Faculty of Life and Health Sciences, Huzhou College, Huzhou, China; 2 School of Public Health, Zhejiang Chinese Medical University, Hangzhou, China; 3 Huzhou Hospital of Traditional Chinese Medicine Affiliated to Zhejiang Chinese Medical University, Hangzhou, China; 4 The Second Clinical Medical College, Zhejiang Chinese Medical University, Hangzhou, China; 5 School of Basic Medical Science, Zhejiang Chinese Medical University, Hangzhou, China

**Keywords:** artificial intelligence, explainable artificial intelligence, functional recovery, ischemic stroke, machine learning, precision rehabilitation, risk stratification

## Abstract

**Background:**

Functional recovery after ischemic stroke varies substantially across patients and stages of care. Conventional rehabilitation planning relies mainly on clinical scales and physician judgment, which may not fully capture the nonlinear interactions among neurological severity, functional status, comorbidities, treatment exposure, and rehabilitation stage. This study aimed to develop and internally validate an artificial intelligence-driven precision rehabilitation model for predicting poor rehabilitation outcome across the continuum of care in patients with ischemic stroke.

**Methods:**

In this retrospective cohort study, 268 patients with ischemic stroke were divided into a training cohort (*n* = 188) and an internal validation cohort (*n* = 80) using stratified random sampling to preserve the outcome distribution. Candidate predictors included demographic characteristics, vascular risk factors, comorbidity burden, baseline National Institutes of Health Stroke Scale (NIHSS) score, baseline Modified Barthel Index (MBI) score, rehabilitation stage, and treatment-related variables. Poor rehabilitation outcome was defined as an MBI score below 85 or a reduction in NIHSS score of less than 2 points during the observation period. Logistic regression, random forest, extreme gradient boosting (XGBoost), and Light Gradient Boosting Machine models were developed and compared. Model performance was evaluated using discrimination, calibration, decision curve analysis, and SHapley Additive exPlanations.

**Results:**

Poor rehabilitation outcome occurred in 104 patients (38.8%). In the internal validation cohort, XGBoost showed the best overall performance, with an area under the receiver operating characteristic curve of 0.862 (95% CI, 0.776–0.923), accuracy of 0.813, sensitivity of 0.806 (95% CI, 0.625–0.926), specificity of 0.816 (95% CI, 0.679–0.912), F1 score of 0.781, and Brier score of 0.139. Calibration analysis showed acceptable agreement between predicted and observed risks. Decision curve analysis indicated greater net clinical benefit than treat-all or treat-none strategies across threshold probabilities of 0.20–0.75. SHAP analysis identified baseline MBI score, baseline NIHSS score, age, structured rehabilitation therapy, rehabilitation stage, and comorbidity burden as the most influential predictors. Model-derived low-, intermediate-, and high-risk groups showed poor outcome rates of 12.8%, 34.4%, and 68.6%, respectively.

**Conclusion:**

The proposed artificial intelligence-driven model showed favorable discrimination, acceptable calibration, and clinically meaningful risk stratification for ischemic stroke rehabilitation. It may provide a preliminary framework for individualized rehabilitation risk stratification across the continuum of care; however, external multicenter validation and prospective clinical evaluation are required before routine clinical application.

## 1 Introduction

Ischemic stroke remains a major cause of mortality and long-term disability worldwide, placing a substantial burden on patients, families, and healthcare systems (1, 2). Although advances in acute stroke management, including reperfusion therapy and organized stroke-unit care, have improved early survival, many survivors continue to experience persistent neurological deficits, impaired activities of daily living, and reduced social participation (3, 4). For these patients, rehabilitation is not simply an adjunctive treatment after acute care, but a core component of long-term functional recovery and secondary prevention.

Functional recovery after ischemic stroke is highly heterogeneous. Patients with comparable neurological impairment at admission may follow markedly different recovery trajectories, ranging from rapid improvement to prolonged functional limitation or chronic dependency (4, 5). This variability reflects the combined influence of age, stroke severity, baseline functional status, vascular risk factors, comorbidity burden, rehabilitation timing, treatment intensity, and individual biological reserve (5, 6). Clinical scales such as the National Institutes of Health Stroke Scale and the Modified Barthel Index are widely used to evaluate neurological impairment and functional independence, but they provide only partial information regarding future recovery potential. In routine clinical practice, rehabilitation planning still relies heavily on clinician experience and stage-based judgment, which may be insufficient for individualized decision-making.

The concept of precision rehabilitation has emerged to address this limitation. Rather than applying uniform rehabilitation strategies to broad patient groups, precision rehabilitation aims to match the intensity, content, and follow-up schedule of rehabilitation to each patient's risk profile and expected recovery trajectory (6, 7). This approach is particularly relevant in ischemic stroke because recovery determinants are not static. In the acute stage, neurological severity, early medical stabilization, vascular risk control, and early mobilization may be especially important (3, 8). During the recovery stage, rehabilitation intensity, functional reserve, adherence, and neuroplasticity-related factors become increasingly relevant (4, 7). In the chronic or sequelae stage, residual disability, frailty, recurrent vascular risk, and access to community-based rehabilitation may have greater prognostic value (6, 9). Therefore, a clinically useful model should not be restricted to a single time point, but should support risk estimation across the continuum of care.

Traditional statistical models have contributed to identifying prognostic factors after stroke, but many existing models focus on mortality, recurrence, discharge destination, or general functional outcome rather than rehabilitation response (10, 11). Moreover, regression-based approaches may be limited when complex nonlinear associations or interactions exist among clinical variables. Recent systematic reviews have shown growing interest in machine learning for predicting post-stroke rehabilitation outcomes, but they have also highlighted several challenges, including limited sample sizes, inconsistent validation, high risk of bias, and insufficient clinical interpretability (10–12). These limitations restrict the translation of predictive models into rehabilitation decision support.

Artificial intelligence and machine learning provide an opportunity to improve individualized recovery prediction. Ensemble algorithms such as random forest, extreme gradient boosting, and Light Gradient Boosting Machine can accommodate mixed clinical data types and identify nonlinear interactions without requiring strict assumptions regarding predictor-outcome relationships (13–15, 31, 32). However, flexible machine learning models may be susceptible to overfitting, particularly when applied to small or medium-sized clinical datasets. Therefore, in the present study, feature selection, five-fold cross-validation, hyperparameter tuning, regularization, and internal validation were incorporated into the modeling strategy to improve model stability and reduce optimism in performance estimation. Recent studies suggest that machine learning models may improve post-stroke functional outcome prediction when demographic, clinical, functional, imaging, and rehabilitation-related variables are integrated (11, 12, 16). However, predictive accuracy alone is not sufficient for clinical implementation. A model intended for rehabilitation planning must also demonstrate acceptable calibration, clinical usefulness, and transparent reporting according to contemporary prediction-model standards (17, 33–35).

Interpretability is particularly important in rehabilitation medicine. A black-box model that provides only a risk score may be difficult for clinicians, therapists, patients, and caregivers to accept. Explainable artificial intelligence methods, such as SHapley Additive exPlanations, can quantify the contribution of individual predictors and clarify how specific variables influence model output (18). This allows clinicians to determine whether a high predicted risk of poor recovery is primarily driven by advanced age, severe baseline neurological deficit, low functional independence, comorbidity burden, inadequate rehabilitation exposure, or other potentially modifiable factors. In this way, explainable artificial intelligence may help transform prediction results into clinically meaningful rehabilitation strategies.

Another important feature of real-world stroke rehabilitation is the coexistence of multiple treatment modalities. In addition to standard medical therapy and physical rehabilitation, patients may receive acupuncture, traditional Chinese medicine-based interventions, and other integrative therapies in some clinical settings. Previous evidence suggests that acupuncture combined with rehabilitation may improve neurological function and activities of daily living in selected patients with ischemic stroke, although the quality and heterogeneity of evidence should be carefully considered (9). Traditional Chinese medicine syndrome patterns may also reflect symptom clusters, constitutional status, and stage-related clinical characteristics that are not fully captured by conventional neurological scales. When appropriately encoded as candidate predictors, these variables may enrich patient phenotyping without changing the primary international framework of precision rehabilitation.

Despite these advances, several gaps remain. First, most prediction models were designed for general stroke prognosis rather than rehabilitation-oriented decision-making. Second, few models explicitly consider the continuum from acute care to recovery and chronic rehabilitation. Third, treatment-related and integrative rehabilitation variables are not consistently incorporated. Fourth, the calibration, clinical net benefit, and interpretability of many models remain insufficiently evaluated. Addressing these gaps may help clinicians identify patients at high risk of poor recovery, allocate rehabilitation resources more efficiently, and develop individualized management strategies.

Therefore, this study aimed to develop and internally validate an artificial intelligence-driven precision rehabilitation model for ischemic stroke across the continuum of care. By integrating demographic characteristics, neurological severity, baseline functional status, vascular risk factors, comorbidities, rehabilitation-stage information, and treatment-related variables, we sought to construct an interpretable model for predicting poor rehabilitation outcome. Multiple algorithms, including conventional logistic regression and machine learning models, were compared in terms of discrimination, calibration, and clinical net benefit. Explainable artificial intelligence analysis was further applied to identify key predictors and support individualized risk stratification for stage-sensitive rehabilitation planning.

## 2 Materials and methods

### 2.1 Study design and oversight

This retrospective cohort study was designed to develop and internally validate an artificial intelligence-driven precision rehabilitation model for predicting poor rehabilitation outcome in patients with ischemic stroke. The study was conducted using routinely collected clinical data from hospitalized patients who underwent medical treatment and rehabilitation management after ischemic stroke. The overall study design, predictor selection, model development, internal validation, and reporting strategy followed contemporary recommendations for clinical prediction models using regression and machine learning approaches (17).

The study protocol was reviewed and approved by the institutional ethics committee. Because the study was based on anonymized retrospective clinical data, the requirement for written informed consent was waived. All patient-level information was de-identified before analysis, and the study was conducted in accordance with the principles of the Declaration of Helsinki.

### 2.2 Study population

Patients with ischemic stroke who received inpatient neurological care and rehabilitation assessment were screened for eligibility. A total of 268 patients were included in the final analysis. The cohort was intended to represent patients across the continuum of post-stroke care, including the acute stage, recovery stage, and chronic or sequelae stage. This design allowed the prediction model to incorporate stage-related differences in functional status, treatment exposure, and rehabilitation response.

Ischemic stroke was diagnosed according to clinical manifestations and neuroimaging findings, including computed tomography or magnetic resonance imaging. Baseline neurological and functional assessments were performed at admission or at the beginning of the rehabilitation episode. Treatment-related variables were extracted from medical and rehabilitation records during hospitalization or structured rehabilitation management.

### 2.3 Inclusion and exclusion criteria

#### 2.3.1 Inclusion criteria

1) age 18 years or older; 2) confirmed diagnosis of ischemic stroke; 3) available baseline neurological evaluation using the National Institutes of Health Stroke Scale; 4) available functional assessment using the Modified Barthel Index; 5) documented treatment and rehabilitation information; and 6) available follow-up or discharge data for outcome assessment.

#### 2.3.2 Exclusion criteria

1) hemorrhagic stroke, transient ischemic attack without confirmed infarction, or nonvascular neurological disease; 2) severe pre-existing disability unrelated to the index stroke that substantially affected activities of daily living before stroke onset; 3) severe systemic disease, malignant tumor, or end-stage organ failure limiting rehabilitation participation; 4) recurrent stroke during the observation period; 5) incomplete key outcome data; or 6) missing baseline variables that could not be reliably recovered from medical records.

### 2.4 Definition of rehabilitation stages

To reflect the continuum of care after ischemic stroke, patients were classified into three rehabilitation stages according to the time from stroke onset to the index rehabilitation assessment. The acute stage was defined as within 1 month after stroke onset. The recovery stage was defined as 1–6 months after onset. The chronic or sequelae stage was defined as more than 6 months after onset. This stage classification was used as both a descriptive variable and a candidate predictor in the model, because the determinants of recovery may differ across the early, intermediate, and long-term phases after stroke.

### 2.5 Study scheme

The study was conducted in six sequential steps. First, eligible patients were identified according to predefined inclusion and exclusion criteria. Second, demographic, clinical, neurological, functional, treatment-related, and rehabilitation-stage variables were extracted from medical records. Third, data preprocessing was performed, including data cleaning, missing-value handling, categorical-variable encoding, and consistency checks. Fourth, the cohort was divided into a training cohort and an internal validation cohort using stratified random sampling. Fifth, conventional logistic regression and machine learning models were developed and compared. Sixth, the best-performing model was interpreted using explainable artificial intelligence and converted into a risk-stratified precision rehabilitation framework.

### 2.6 Outcome definition

The primary outcome was poor rehabilitation outcome. This outcome was defined as failure to achieve adequate functional recovery at follow-up or discharge. Specifically, poor rehabilitation outcome was defined as a Modified Barthel Index score below 85 or an improvement in National Institutes of Health Stroke Scale score of less than 2 points during the observation period. Patients who achieved functional independence or clinically meaningful neurological improvement were classified as having a favorable rehabilitation outcome.

The MBI component was selected because independence in activities of daily living is a central target of stroke rehabilitation. An MBI score below 85 was used to indicate that the patient had not achieved satisfactory functional independence during the observation period. The NIHSS component was included to capture neurological improvement, and an NIHSS reduction of less than 2 points was considered to represent limited neurological recovery. This composite endpoint was therefore intended to reflect both functional independence and neurological change, rather than relying on a single dimension of recovery.

### 2.7 Candidate predictors

Candidate predictors were selected before model development according to clinical relevance, previous evidence, and routine availability in rehabilitation practice. Predictors were grouped into five domains.

The first domain included demographic characteristics: age, sex, educational level, smoking history, and alcohol consumption. The second domain included vascular risk factors and clinical comorbidities: hypertension, diabetes mellitus, atrial fibrillation, coronary heart disease, previous stroke history, carotid plaque, and comorbidity burden. Comorbidity burden was defined according to the number of major vascular or systemic diseases and was categorized as fewer than two comorbidities or two or more comorbidities.

The third domain included neurological and functional variables: baseline National Institutes of Health Stroke Scale score and baseline Modified Barthel Index score. The fourth domain included treatment-related variables: antiplatelet therapy, anticoagulant therapy, statin therapy, antihypertensive therapy, glucose-lowering therapy, structured rehabilitation therapy, acupuncture therapy, and traditional Chinese medicine-based treatment. These variables were coded according to actual treatment exposure during the observation period.

The fifth domain included traditional Chinese medicine-related clinical features when available. Syndrome patterns were encoded as categorical variables, including excess-dominant patterns, deficiency-dominant patterns, phlegm-stasis-related patterns, and qi-yin deficiency with blood stasis-related patterns. These variables were not used to redefine the disease phenotype, but were incorporated as candidate predictors to enrich real-world patient characterization within the broader framework of precision rehabilitation.

### 2.8 Data preprocessing

All variables were examined for completeness, plausibility, and internal consistency before analysis. Continuous variables were checked for extreme values and distributional characteristics. Values that were clinically implausible were verified against the original medical records when possible. Categorical variables were checked for coding consistency and category frequency.

Variables with excessive missingness were excluded from model development. For variables with limited missingness, imputation was performed within the training cohort to prevent information leakage. The missing rates of key variables were low, ranging from 0% to 4.9%. Specifically, missingness was 0% for age, sex, stroke stage, baseline NIHSS score, baseline MBI score, and primary outcome; 1.1% for smoking history; 1.5% for alcohol consumption; 2.2% for carotid plaque; 2.6% for comorbidity burden; 3.0% for rehabilitation therapy; 3.4% for acupuncture therapy; and 4.9% for traditional Chinese medicine-related variables. Because no key predictor had a missing rate above 5%, continuous variables were imputed using median values and categorical variables using the most frequent category. This simple imputation strategy was selected to preserve sample size while avoiding excessive model complexity in a moderate-sized cohort. The same imputation parameters derived from the training cohort were applied to the internal validation cohort. Categorical variables were transformed using one-hot encoding. Rare categories were combined when clinically appropriate to reduce sparse feature representation.

Continuous variables were standardized when required by a specific algorithm. Tree-based models were trained using the original scale of the variables because these algorithms are generally insensitive to monotonic transformations. All preprocessing steps were fitted only on the training cohort and then applied to the validation cohort.

### 2.9 Model development

The full cohort was randomly divided into a training cohort and an internal validation cohort at a ratio of 7:3. Stratified random sampling was applied to maintain a similar proportion of poor rehabilitation outcomes in both cohorts. The training cohort was used for feature selection, model fitting, and hyperparameter tuning. The validation cohort was reserved for final evaluation of model performance.

Four prediction models were developed: logistic regression, random forest, extreme gradient boosting, and Light Gradient Boosting Machine. Logistic regression was used as the conventional reference model because of its interpretability and widespread use in clinical prediction research. Random forest, XGBoost, and LightGBM were selected because these ensemble-learning algorithms can capture nonlinear associations and complex interactions among clinical variables (13–15).

Feature selection was conducted exclusively within the training cohort to avoid information leakage. Candidate predictors were first screened according to clinical relevance and routine availability. LASSO regression and model-based feature-importance ranking were then used to reduce redundant predictors. Hyperparameters were optimized using five-fold cross-validation in the training cohort. For random forest, the number of trees, maximum tree depth, minimum samples per split, and number of variables considered at each split were tuned. For XGBoost and LightGBM, the learning rate, number of estimators, maximum depth, subsampling ratio, column sampling ratio, and regularization parameters were optimized. Early stopping was applied where appropriate. These procedures were used to improve model stability and reduce the risk of overfitting.

To reduce the risk of overfitting, candidate predictors were limited to clinically relevant variables, feature selection was performed within the training cohort, and hyperparameter tuning was conducted using five-fold cross-validation. Model complexity was further controlled by regularization, tree-depth restriction, subsampling, and early stopping where applicable. The internal validation cohort was reserved exclusively for final model evaluation.

### 2.10 Internal validation and model performance

Model performance was evaluated in the internal validation cohort. Discrimination was assessed using the area under the receiver operating characteristic curve. Accuracy, sensitivity, specificity, positive predictive value, negative predictive value, and F1 score were also calculated. Ninety-five percent confidence intervals for the area under the receiver operating characteristic curve were estimated using bootstrap resampling. Differences in discrimination between models were compared using the DeLong test (19).

Calibration was evaluated using calibration plots, calibration intercept, calibration slope, and Brier score. A calibration curve was generated to compare predicted probabilities with observed outcome frequencies across risk strata. A calibration slope close to 1.0 and a lower Brier score were considered indicators of better probabilistic prediction.

Clinical usefulness was assessed using decision curve analysis (20). Net benefit was calculated across a range of clinically relevant threshold probabilities to determine whether the model provided greater benefit than treat-all or treat-none strategies. The final model was selected based on overall performance, including discrimination, calibration, clinical net benefit, and interpretability.

### 2.11 Explainable artificial intelligence analysis

To improve clinical interpretability, SHapley Additive exPlanations analysis was applied to the best-performing machine learning model (18). SHAP summary plots were generated to rank the importance of predictors and visualize the direction of their effects on predicted risk. Mean absolute SHAP values were used to quantify the global contribution of each predictor.

SHAP dependence plots were planned for key continuous variables, including age, baseline National Institutes of Health Stroke Scale score, and baseline Modified Barthel Index score. Individual-level SHAP explanations were also generated for representative patients in different risk categories to illustrate how the model combined neurological severity, functional status, disease stage, comorbidity burden, and treatment exposure to estimate individualized risk.

### 2.12 Risk stratification and precision rehabilitation framework

Predicted probabilities generated by the final model were used to stratify patients into three risk groups. Patients with predicted probabilities below 0.30 were classified as low risk, those with probabilities between 0.30 and 0.60 were classified as intermediate risk, and those with probabilities above 0.60 were classified as high risk. The observed proportion of poor rehabilitation outcomes was compared across these groups to evaluate the clinical relevance of the stratification strategy.

A precision rehabilitation framework was then developed according to model-derived risk categories. Low-risk patients were considered suitable for standard rehabilitation and routine follow-up. Intermediate-risk patients were considered candidates for intensified rehabilitation, closer functional reassessment, and timely adjustment of treatment intensity. High-risk patients were considered to require multidisciplinary rehabilitation planning, closer monitoring, early optimization of secondary prevention, family education, and transition planning for community-based rehabilitation.

### 2.13 Sensitivity analyses

Several sensitivity analyses were performed to examine the robustness of the model. First, model development was repeated after excluding traditional Chinese medicine-related variables to evaluate their incremental contribution. Second, an alternative outcome definition based only on Modified Barthel Index improvement was tested. Third, model performance was assessed separately in acute, recovery, and chronic-stage subgroups. Fourth, repeated bootstrap resampling was used to evaluate the stability of selected predictors and model performance. In addition, a sensitivity analysis was performed using multivariate imputation by chained equations to assess whether the primary imputation strategy influenced model performance.

### 2.14 Statistical analysis

Continuous variables were summarized as mean and standard deviation or median and interquartile range, depending on distribution. Categorical variables were summarized as counts and percentages. Baseline characteristics between the training and validation cohorts were compared using Student's t-test or the Mann–Whitney U test for continuous variables and the chi-square test or Fisher's exact test for categorical variables. Comparisons between patients with favorable and poor rehabilitation outcomes were performed using the same approach.

Multivariable logistic regression was used to estimate the independent associations between candidate predictors and poor rehabilitation outcome. Odds ratios and 95% confidence intervals were reported. For all statistical tests, a two-sided *P* value less than 0.05 was considered statistically significant. All analyses were conducted using R and Python. Machine learning models were developed using standard packages, including scikit-learn, XGBoost, LightGBM, and SHAP.

## 3 Results

### 3.1 Study cohort and outcome distribution

**Figure 1:**
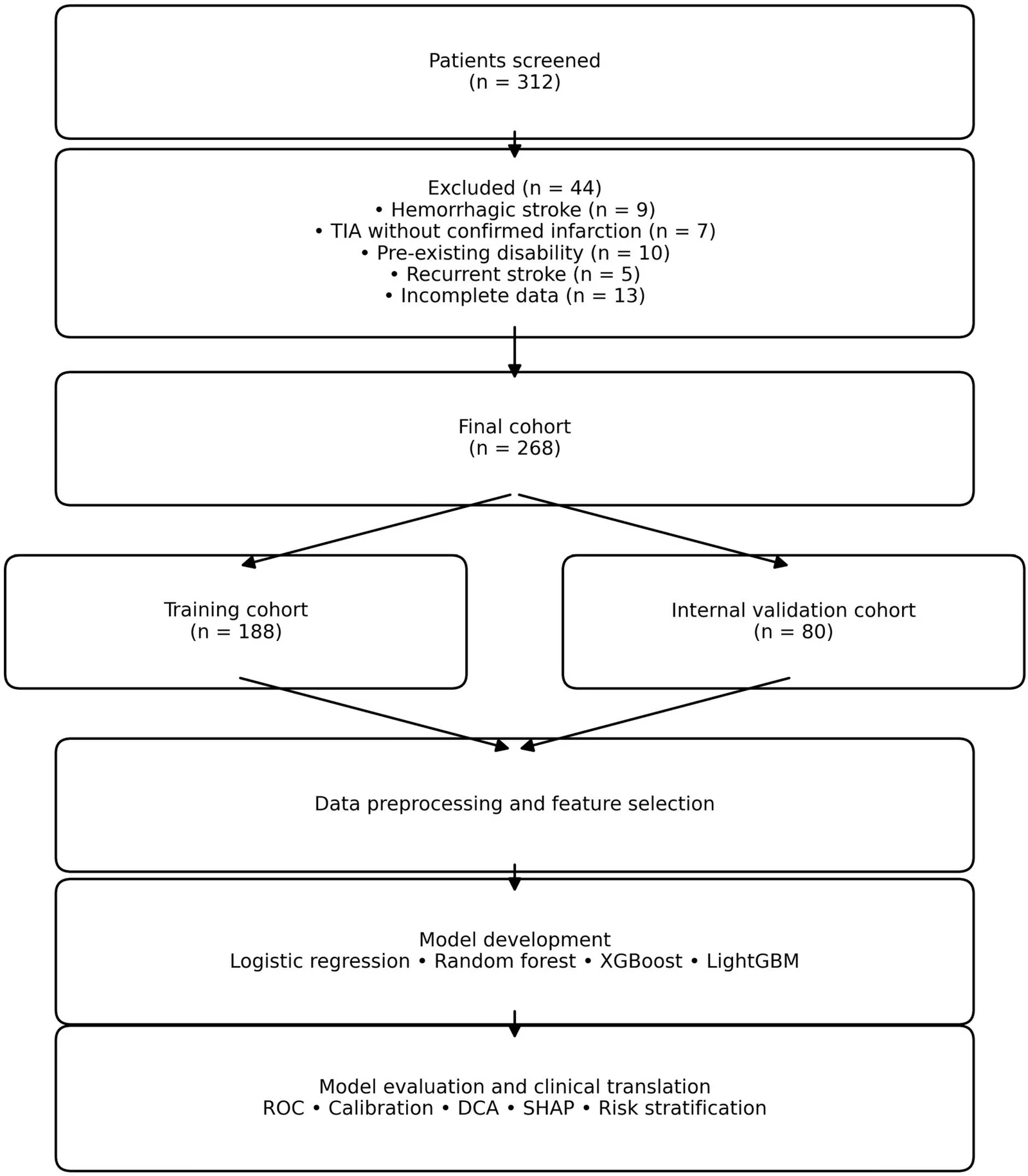
Study workflow and model development pipeline. The figure illustrates patient screening, cohort construction, data splitting, model development, internal validation, explainable artificial intelligence analysis, and construction of the risk-stratified precision rehabilitation framework.

A total of 312 patients with ischemic stroke were initially screened. After applying the predefined eligibility criteria, 44 patients were excluded, including 9 with hemorrhagic stroke, 7 with transient ischemic attack without confirmed infarction, 10 with severe pre-existing disability, 5 with recurrent stroke during the observation period, and 13 with incomplete key clinical or outcome data. Finally, 268 patients were included in the analysis. Among them, 188 patients were assigned to the training cohort and 80 patients to the internal validation cohort using stratified random sampling (Figure 1).

Across the whole cohort, 112 patients (41.8%) were in the acute stage, 96 (35.8%) were in the recovery stage, and 60 (22.4%) were in the chronic or sequelae stage. A favorable rehabilitation outcome was observed in 164 patients (61.2%), whereas 104 patients (38.8%) met the definition of poor rehabilitation outcome. The proportion of poor rehabilitation outcome was comparable between the training and validation cohorts (38.8% vs. 38.8%, *P* = 0.994), indicating adequate preservation of outcome distribution after data splitting.

### 3.2 Baseline characteristics of the training and validation cohorts

The baseline characteristics of the training and validation cohorts are shown in Table 1. The mean age was 69.8 ± 11.6 years in the training cohort and 70.4 ± 12.0 years in the validation cohort. Male patients accounted for 54.3% and 52.5% of the training and validation cohorts, respectively. The distributions of rehabilitation stages were similar between the two cohorts, with acute-stage patients accounting for 42.0% in the training cohort and 41.3% in the validation cohort.

**Table 1:** Baseline characteristics of the training and internal validation cohorts.

Variable	Training cohort (*n* = 188)	Validation cohort (*n* = 80)	*P* value
Age, years	69.8 ± 11.6	70.4 ± 12.0	0.694
Male sex, *n* (%)	102 (54.3)	42 (52.5)	0.789
Acute stage, *n* (%)	79 (42.0)	33 (41.3)	0.914
Recovery stage, *n* (%)	67 (35.6)	29 (36.3)	0.92
Chronic/sequelae stage, *n* (%)	42 (22.3)	18 (22.5)	0.976
Baseline NIHSS score	5.2 ± 3.1	5.5 ± 3.4	0.482
Baseline MBI score	67.4 ± 18.6	66.1 ± 19.3	0.604
Hypertension, *n* (%)	121 (64.4)	50 (62.5)	0.771
Diabetes mellitus, *n* (%)	55 (29.3)	24 (30.0)	0.908
Atrial fibrillation, *n* (%)	29 (15.4)	13 (16.3)	0.864
Smoking history, *n* (%)	62 (33.0)	28 (35.0)	0.749
Carotid plaque, *n* (%)	109 (58.0)	47 (58.8)	0.907
Comorbidity burden ≥2, *n* (%)	74 (39.4)	33 (41.3)	0.775
Rehabilitation therapy, *n* (%)	149 (79.3)	61 (76.3)	0.581
Acupuncture therapy, *n* (%)	109 (58.0)	44 (55.0)	0.652
Traditional Chinese medicine therapy, *n* (%)	98 (52.1)	41 (51.3)	0.899
Antiplatelet therapy, *n* (%)	154 (81.9)	66 (82.5)	0.912
Statin therapy, *n* (%)	139 (73.9)	60 (75.0)	0.854
Poor rehabilitation outcome, *n* (%)	73 (38.8)	31 (38.8)	0.994

Values are shown as mean ± standard deviation or *n* (%).

NIHSS, National Institutes of Health Stroke Scale; MBI, Modified Barthel Index.

Baseline neurological impairment and functional status were also well balanced. The mean baseline NIHSS score was 5.2 ± 3.1 in the training cohort and 5.5 ± 3.4 in the validation cohort (*P* = 0.482). The mean baseline MBI score was 67.4 ± 18.6 and 66.1 ± 19.3, respectively (*P* = 0.604). No statistically significant differences were observed between cohorts in vascular risk factors, comorbidity burden, carotid plaque, rehabilitation therapy, acupuncture therapy, traditional Chinese medicine-based treatment, antiplatelet therapy, or statin therapy. These findings suggested that the internal validation cohort was representative of the training cohort.

### 3.3 Clinical characteristics associated with poor rehabilitation outcome

Compared with patients who achieved favorable rehabilitation outcomes, those with poor rehabilitation outcomes were older, had more severe neurological impairment, and showed lower baseline functional independence. The mean age was 75.1 ± 11.8 years in the poor-outcome group and 66.8 ± 10.7 years in the favorable-outcome group (*P* < 0.001). Baseline NIHSS score was significantly higher in the poor-outcome group (7.4 ± 3.5 vs. 3.9 ± 2.4, *P* < 0.001), whereas baseline MBI score was markedly lower (52.8 ± 18.7 vs. 76.9 ± 13.8, *P* < 0.001).

Stage distribution also differed between outcome groups. Chronic or sequelae-stage patients accounted for 33.7% of the poor-outcome group but only 15.2% of the favorable-outcome group (*P* < 0.001). Patients with poor outcomes were more likely to have hypertension (72.1% vs. 58.5%, *P* = 0.026), diabetes mellitus (37.5% vs. 24.4%, *P* = 0.021), atrial fibrillation (23.1% vs. 11.0%, *P* = 0.007), carotid plaque (69.2% vs. 51.2%, *P* = 0.004), and a comorbidity burden of two or more diseases (53.8% vs. 31.1%, *P* < 0.001).

Treatment exposure differed between groups. Structured rehabilitation therapy was less frequent in patients with poor outcomes than in those with favorable outcomes (69.2% vs. 84.1%, *P* = 0.004). Similar patterns were observed for acupuncture therapy (47.1% vs. 63.4%, *P* = 0.009) and traditional Chinese medicine-based treatment (43.3% vs. 57.3%, *P* = 0.026). These findings indicated that both baseline disease severity and treatment-related factors were associated with rehabilitation prognosis.

In multivariable logistic regression analysis, older age, higher baseline NIHSS score, lower baseline MBI score, chronic-stage status, and comorbidity burden of two or more diseases remained independently associated with poor rehabilitation outcome. Structured rehabilitation therapy was independently associated with a reduced likelihood of poor outcome. Acupuncture therapy and traditional Chinese medicine-based treatment showed protective trends, although their independent associations did not reach statistical significance after adjustment for neurological severity and functional status.

### 3.4 Feature selection and final predictors

Feature selection was performed in the training cohort using clinical relevance, LASSO regression, and model-based feature-importance ranking. The optimal LASSO penalty parameter was selected by five-fold cross-validation, and 13 variables were retained for model development. These variables included age, baseline NIHSS score, baseline MBI score, rehabilitation stage, comorbidity burden, carotid plaque, diabetes mellitus, atrial fibrillation, hypertension, structured rehabilitation therapy, acupuncture therapy, traditional Chinese medicine-based treatment, and traditional Chinese medicine syndrome pattern.

**Figure 2:**
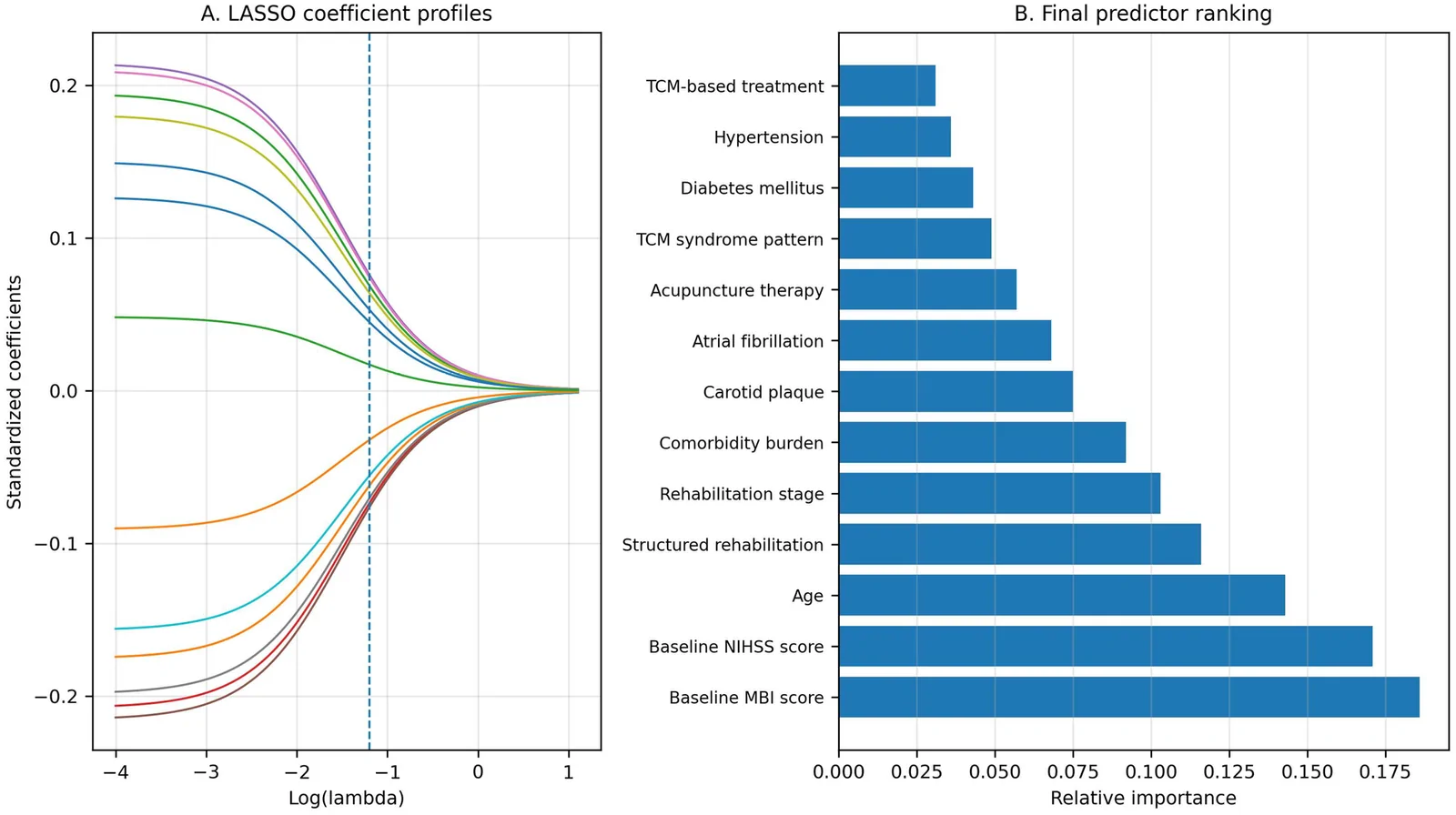
Feature selection and final predictor ranking. Panel **(A)** shows the LASSO coefficient profiles. Panel **(B)** shows the model-derived relative importance of the final predictors retained for model development.

The retained predictors reflected five clinically meaningful dimensions: demographic vulnerability, neurological severity, baseline functional reserve, vascular and systemic disease burden, and rehabilitation-related treatment exposure. Baseline MBI score, baseline NIHSS score, age, rehabilitation stage, and comorbidity burden ranked consistently among the most informative variables across feature-selection approaches (Figure 2).

### 3.5 Model performance in the internal validation cohort

**Figure 3:**
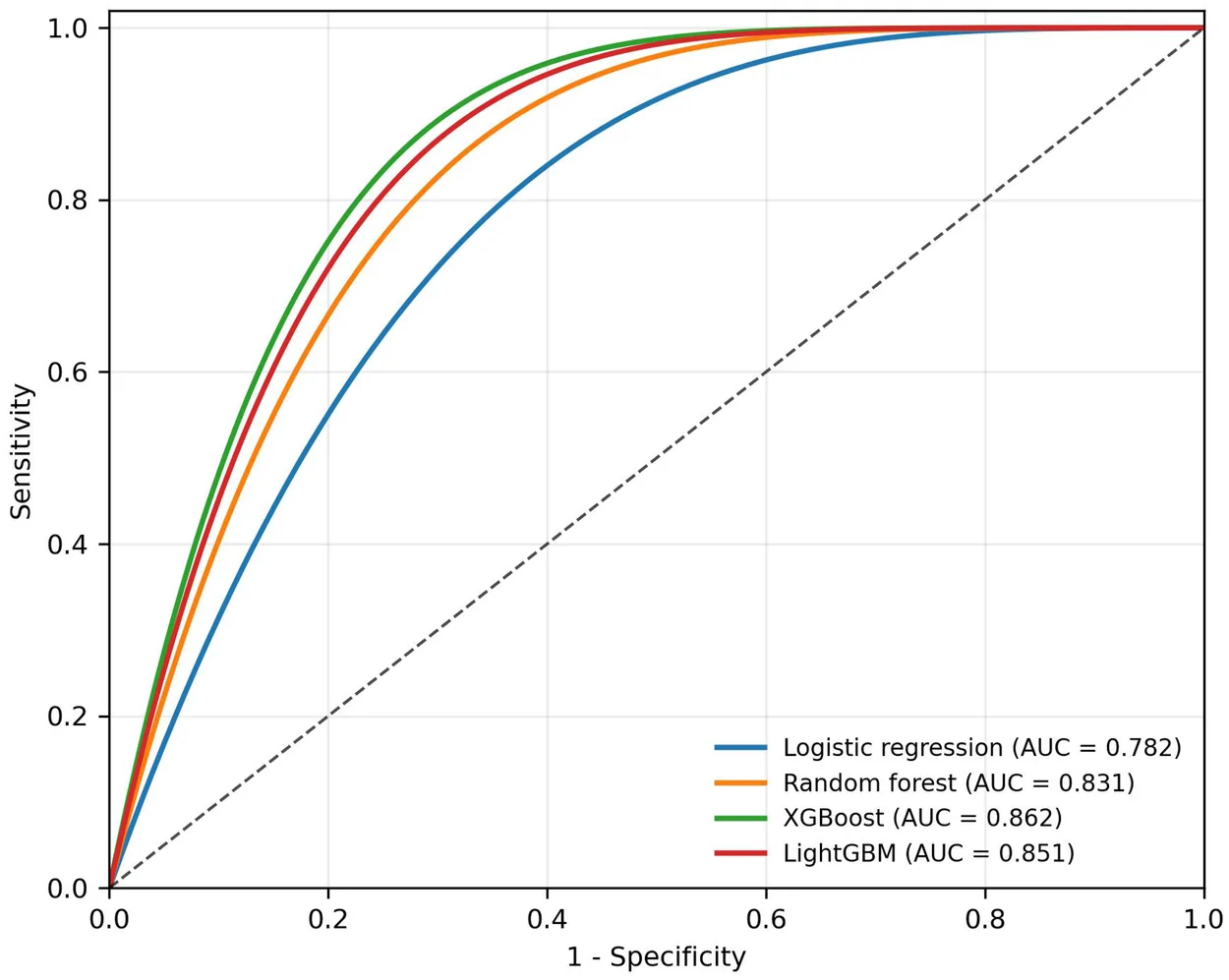
Receiver operating characteristic curves of the four prediction models in the internal validation cohort. XGBoost showed the highest discrimination and significantly outperformed logistic regression according to the DeLong test (*P* = 0.035).

The performance of the four prediction models in the internal validation cohort is summarized in Table 2 and Figure 3. All models demonstrated acceptable discrimination for predicting poor rehabilitation outcome. Among them, the XGBoost model achieved the highest AUC of 0.862 (95% CI, 0.776–0.923), followed by LightGBM with an AUC of 0.851 (95% CI, 0.761–0.914), random forest with an AUC of 0.831 (95% CI, 0.739–0.899), and logistic regression with an AUC of 0.782 (95% CI, 0.681–0.862).

**Table 2:** Performance comparison of prediction models in the internal validation cohort.

Model	AUC	95% CI	Accuracy	Sensitivity	Specificity	F1 score	Brier score
Logistic regression	0.782	0.681–0.862	0.738	0.71	0.755	0.71	0.178
Random forest	0.831	0.739–0.899	0.775	0.774	0.776	0.75	0.153
XGBoost	0.862	0.776–0.923	0.813	0.806	0.816	0.781	0.139
LightGBM	0.851	0.761–0.914	0.8	0.774	0.816	0.762	0.145

AUC, area under the receiver operating characteristic curve; CI, confidence interval.

The XGBoost model also showed the highest overall accuracy (0.813), balanced sensitivity (0.806) and specificity (0.816), and the highest F1 score (0.781). The Brier score of XGBoost was 0.139, lower than that of logistic regression, random forest, and LightGBM, indicating better probabilistic prediction. Pairwise comparison using the DeLong test showed that XGBoost had significantly better discrimination than logistic regression (*P* = 0.035). LightGBM also showed better discrimination than logistic regression (*P* = 0.049). However, the differences between XGBoost and random forest (*P* = 0.214) and between XGBoost and LightGBM (*P* = 0.431) were not statistically significant.

Although XGBoost showed the highest AUC and the most favorable overall performance in the internal validation cohort, the absolute differences between XGBoost and the other tree-based models were modest. Given the limited size of the internal validation cohort, the clinical significance of this slight performance advantage remains uncertain. Therefore, XGBoost was selected as the final model for subsequent interpretation based on its overall performance within this dataset, rather than because it was definitively superior to the other tree-based algorithms.

### 3.6 Calibration and clinical utility of the final model

The calibration performance of the XGBoost model was acceptable in the internal validation cohort. The calibration curve showed reasonable agreement between predicted and observed probabilities of poor rehabilitation outcome. The calibration intercept was 0.04, and the calibration slope was 0.92, suggesting no substantial systematic overestimation or underestimation of risk. The Brier score was 0.139, and the expected calibration error was 0.046, further supporting the reliability of the predicted probabilities.

**Figure 4:**
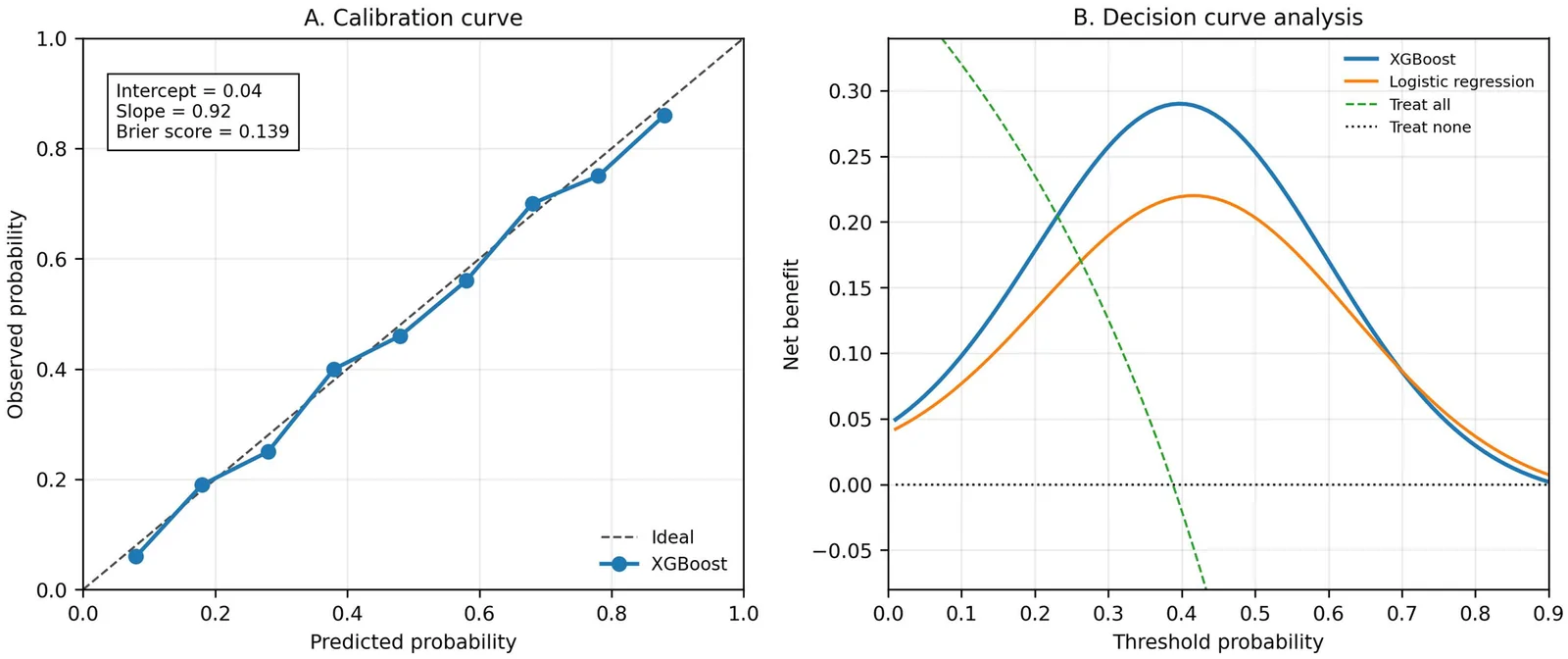
Calibration and clinical utility of the XGBoost model. Panel **(A)** shows the calibration curve. Panel **(B)** shows the decision curve analysis, indicating that the XGBoost model provided greater net benefit than treat-all and treat-none strategies across clinically relevant threshold probabilities.

Decision curve analysis demonstrated that the XGBoost model provided greater net clinical benefit than the treat-all and treat-none strategies across a clinically relevant threshold probability range of 0.20–0.75. The maximum net benefit was 0.29 at a threshold probability of approximately 0.40. These findings suggest that the model may have potential utility for risk-informed rehabilitation stratification in the internal validation cohort. However, this result should be interpreted as preliminary, and external validation is required before the model can be used to guide clinical decision-making in routine practice (Figure 4).

### 3.7 Explainable artificial intelligence analysis

SHAP analysis was performed to interpret the XGBoost model. The global SHAP summary plot showed that baseline MBI score, baseline NIHSS score, age, structured rehabilitation therapy, rehabilitation stage, and comorbidity burden were the most influential predictors. Lower baseline MBI score, higher baseline NIHSS score, older age, chronic-stage status, higher comorbidity burden, carotid plaque, and atrial fibrillation contributed to higher model-predicted risk of poor rehabilitation outcome. In contrast, structured rehabilitation therapy, acupuncture therapy, and traditional Chinese medicine-based treatment were associated with lower model-predicted risk in this dataset.

**Figure 5:**
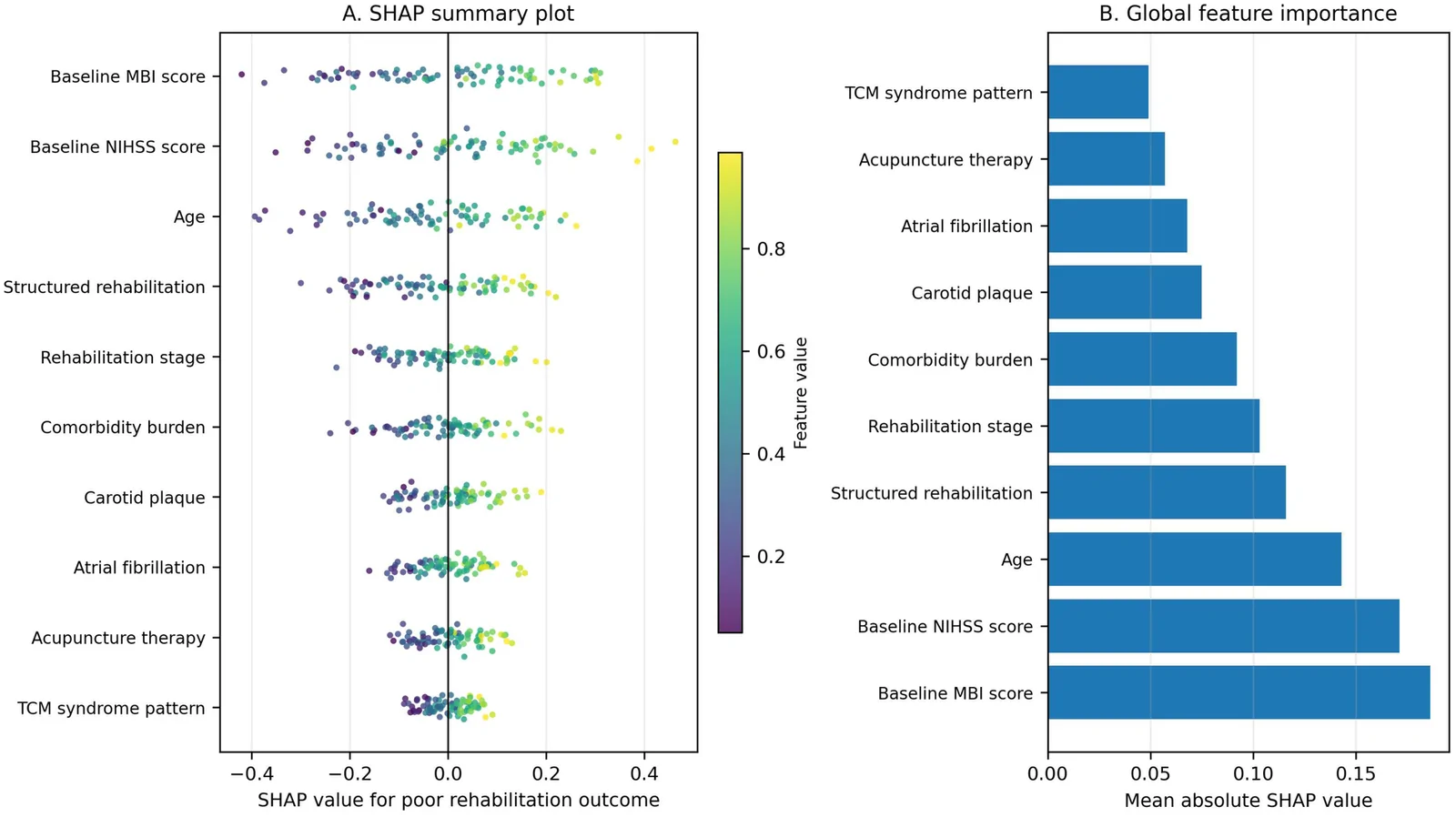
Explainable artificial intelligence analysis of the final model. Panel **(A)** shows the SHAP summary plot. Panel **(B)** shows the ranked mean absolute SHAP values for the most influential predictors.

Higher baseline MBI score showed a negative contribution to model-predicted risk, indicating that patients with better functional independence at baseline were more likely to be classified by the model as having a lower risk of poor rehabilitation outcome. Higher baseline NIHSS score contributed positively to model-predicted risk, reflecting the importance of neurological deficit severity in the prediction process. Age showed a gradual risk-increasing pattern, with older patients having higher predicted probabilities of poor recovery. Structured rehabilitation therapy, acupuncture therapy, and traditional Chinese medicine-based treatment showed inverse predictive associations with poor rehabilitation outcome in the SHAP analysis. These treatment-related findings should be interpreted as predictive associations rather than evidence of causal treatment effects because treatment allocation was observational and may have been affected by confounding by indication (Figure 5).

### 3.8 Risk stratification and precision rehabilitation decision framework

Using the predicted probability generated by the XGBoost model, patients were stratified into low-, intermediate-, and high-risk groups. In the whole cohort, 86 patients were classified as low risk, 96 as intermediate risk, and 86 as high risk. The observed rate of poor rehabilitation outcome increased progressively across the three groups: 12.8% in the low-risk group, 34.4% in the intermediate-risk group, and 68.6% in the high-risk group. The trend was statistically significant (*P* for trend < 0.001).

A similar gradient was observed in the internal validation cohort. Among validation patients, poor rehabilitation outcome occurred in 4 of 26 patients (15.4%) in the low-risk group, 11 of 31 patients (35.5%) in the intermediate-risk group, and 16 of 23 patients (69.6%) in the high-risk group. Compared with the low-risk group, the high-risk group had a markedly increased likelihood of poor rehabilitation outcome, with an estimated odds ratio of 12.6 (95% CI, 3.3–48.1; *P* < 0.001) in the validation cohort.

**Figure 6:**
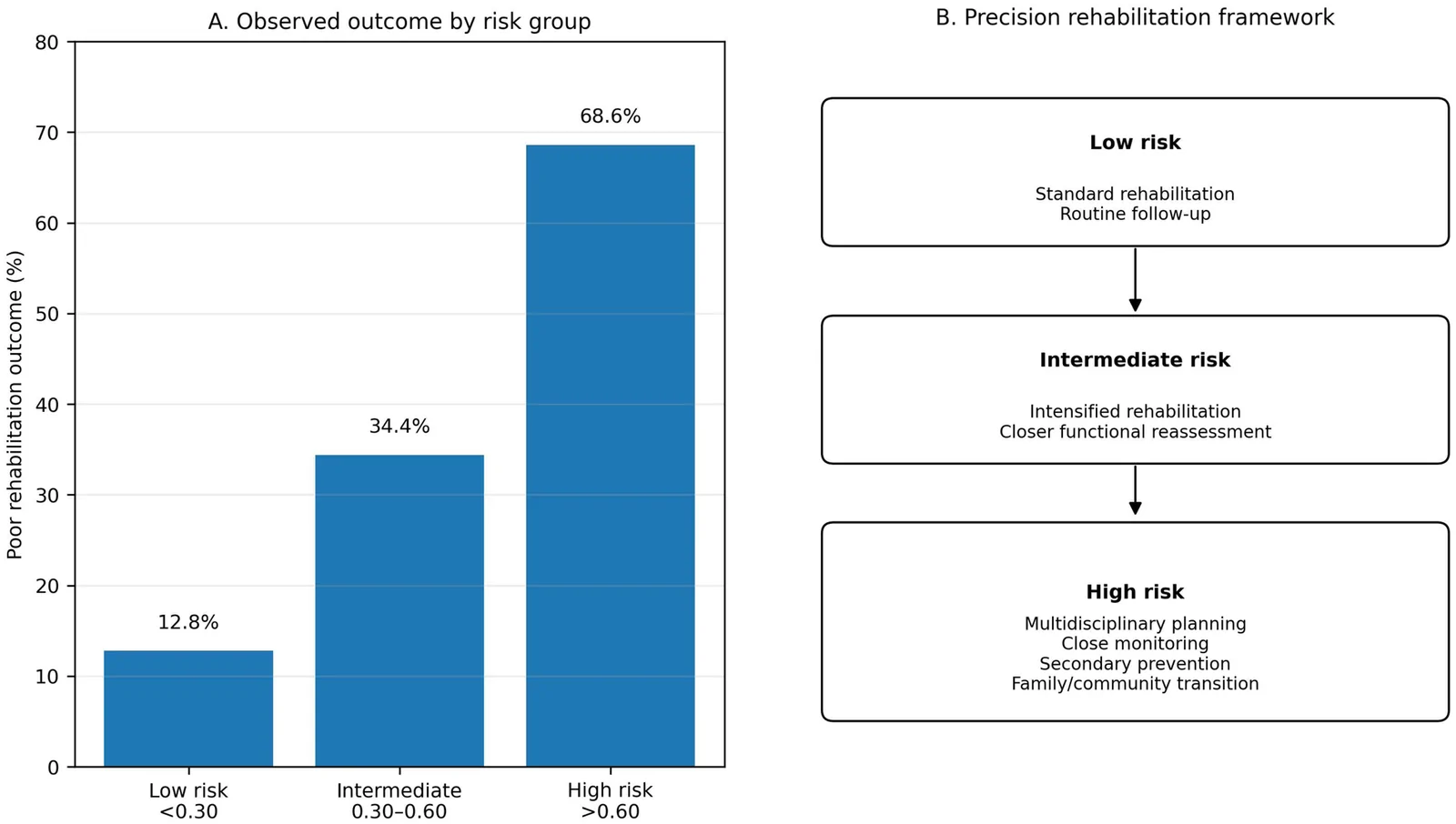
Model-derived risk stratification and precision rehabilitation framework. Panel **(A)** shows the observed rate of poor rehabilitation outcome across low-, intermediate-, and high-risk groups. Panel **(B)** shows the corresponding rehabilitation decision framework.

Based on these risk categories, a precision rehabilitation decision framework was constructed. Low-risk patients were considered suitable for standard rehabilitation and routine follow-up. Intermediate-risk patients were considered candidates for intensified rehabilitation and more frequent functional reassessment. High-risk patients were considered to require multidisciplinary rehabilitation planning, closer neurological and functional monitoring, optimization of secondary prevention, family education, and early transition planning for community-based rehabilitation (Figure 6).

### 3.9 Sensitivity analyses

Sensitivity analyses supported the robustness of the final model. After excluding traditional Chinese medicine-related variables, the AUC of the XGBoost model decreased from 0.862 to 0.844, and the Brier score increased from 0.139 to 0.148. This suggested that traditional Chinese medicine-related variables provided modest incremental information for patient phenotyping, although the core predictive performance remained primarily driven by neurological severity, functional status, age, rehabilitation stage, and comorbidity burden.

When poor rehabilitation outcome was redefined using MBI improvement alone, the XGBoost model maintained acceptable discrimination, with an AUC of 0.846. Subgroup analyses showed that the model performed consistently across rehabilitation stages, with AUC values of 0.858 in the acute stage, 0.841 in the recovery stage, and 0.817 in the chronic or sequelae stage. Bootstrap resampling demonstrated stable selection of baseline MBI score, baseline NIHSS score, age, rehabilitation stage, and structured rehabilitation therapy in more than 80% of repeated samples.

To examine whether the imputation strategy influenced model performance, an additional sensitivity analysis was performed using multivariate imputation by chained equations. The XGBoost model showed similar performance after multivariate imputation, with an AUC of 0.858 and a Brier score of 0.142, compared with an AUC of 0.862 and a Brier score of 0.139 in the primary analysis. These findings suggested that the final model was not materially affected by the imputation method and was not overly dependent on a single outcome definition, a specific stage subgroup, or the inclusion of traditional Chinese medicine-related variables.

## 4 Discussion

In this retrospective cohort study, we developed and internally validated an artificial intelligence-driven precision rehabilitation model to estimate the risk of poor rehabilitation outcome in patients with ischemic stroke across the continuum of care. The principal finding was that a model built on routinely available clinical and rehabilitation information could provide clinically useful individualized risk estimates. Among the candidate algorithms, XGBoost showed the most favorable overall performance, with higher discrimination, lower prediction error, acceptable calibration, and greater net benefit than conventional logistic regression. Beyond model accuracy, the SHAP analysis indicated that the prediction was mainly driven by clinically plausible variables, including baseline functional independence, neurological deficit severity, age, structured rehabilitation exposure, rehabilitation stage, and comorbidity burden. These findings suggest that functional recovery after ischemic stroke should be approached as a dynamic, multidimensional process rather than a fixed consequence of the initial infarct or a single baseline scale.

The importance of baseline MBI and NIHSS in the final model is consistent with the clinical logic of stroke rehabilitation. Baseline MBI reflects the patient's functional reserve at the beginning of the rehabilitation episode, including the ability to perform essential activities of daily living. Patients with low baseline MBI scores are often limited not only by motor impairment but also by impaired balance, self-care dependence, cognitive dysfunction, fatigue, or poor tolerance of intensive training. In contrast, patients with higher functional independence at baseline may have greater residual capacity to engage in task-oriented training and may achieve clinically meaningful improvement more readily. NIHSS provides complementary information by quantifying neurological deficit severity. A higher NIHSS score usually indicates more extensive neurological impairment and is associated with lower independence, increased complication risk, and greater rehabilitation complexity. The fact that these two variables ranked highest in the SHAP analysis supports the construct validity of the model and suggests that the algorithm learned clinically meaningful patterns rather than unstable statistical noise (21–24).

Age was another influential predictor of poor rehabilitation outcome. Older patients may have reduced physiological reserve, lower tolerance for high-intensity rehabilitation, a higher prevalence of sarcopenia and frailty, and more frequent cardiovascular or metabolic comorbidities. Age may also interact with baseline impairment: a moderate neurological deficit in a younger patient and the same deficit in an older patient may lead to different recovery trajectories because of differences in neuroplastic potential, endurance, and home support. Nevertheless, the inclusion of age in the model should not be interpreted as a basis for therapeutic pessimism. Instead, age-sensitive prediction should support realistic goal setting, early caregiver education, prevention of complications, and appropriate tailoring of rehabilitation intensity. A high predicted risk in an older patient should prompt more careful planning rather than reduction of rehabilitation opportunity.

The contribution of rehabilitation stage is also clinically important. Recovery after ischemic stroke does not occur within a single homogeneous time window. In the acute stage, early neurological stability, prevention of complications, mobilization, and secondary prevention are central. In the recovery stage, patients often show the most visible functional gains, and treatment intensity, adherence, neuroplasticity, and task-specific training become increasingly important. In the chronic or sequelae stage, the clinical focus often shifts toward residual disability management, prevention of recurrent events, participation, community reintegration, and long-term self-management. By incorporating acute, recovery, and chronic-stage patients, the present model reflects the clinical reality that rehabilitation decisions are made throughout the care pathway. This continuum-of-care structure distinguishes the present study from prediction models that focus exclusively on discharge outcome or a single post-stroke time point.

The model also identified comorbidity burden and vascular risk factors as relevant contributors to poor recovery. Patients with multiple comorbid conditions are more likely to experience reduced exercise tolerance, medical instability, polypharmacy, recurrent vascular events, and interruptions in rehabilitation. Carotid plaque, atrial fibrillation, diabetes mellitus, and hypertension may represent the broader vascular and metabolic background that shapes both neurological vulnerability and long-term recovery capacity. These findings highlight that stroke rehabilitation cannot be separated from secondary prevention and general medical management. A patient classified as high risk by the model may require not only more intensive physical or occupational therapy but also stricter vascular risk control, nutritional support, fall prevention, and coordinated management of chronic diseases.

Structured rehabilitation therapy was associated with a lower predicted risk of poor outcome and ranked among the most important modifiable variables. Although the retrospective design does not allow causal inference, this finding is consistent with the central role of organized rehabilitation in post-stroke recovery. Importantly, including treatment exposure in the model makes the prediction framework more relevant to clinical decision-making. A model that contains only baseline demographic and disease-severity variables may estimate prognosis, but it offers limited information about modifiable rehabilitation pathways. By incorporating treatment-related variables, the model begins to connect risk prediction with potential intervention strategies. This is essential for precision rehabilitation, where the objective is not simply to identify poor prognosis but to guide stage-appropriate and resource-sensitive management.

The comparison among algorithms showed that tree-based machine learning models outperformed conventional logistic regression, with XGBoost achieving the best overall performance. This result is not unexpected, because post-stroke recovery is influenced by nonlinear relationships and interactions among multiple variables. For example, the effect of baseline MBI may differ according to age, stroke stage, and comorbidity burden; the apparent benefit of rehabilitation exposure may also depend on baseline severity and functional reserve. Logistic regression can be clinically interpretable, but it may underrepresent such complex interactions unless they are specified *a priori*. In contrast, boosting algorithms can model nonlinear structures more flexibly. However, the differences among XGBoost, LightGBM, and random forest were relatively modest, suggesting that the predictive signal was not purely algorithm-dependent. Rather, the selected clinical predictors contained stable prognostic information that could be captured by several tree-based methods. This observation is reassuring because it reduces concern that the reported performance was driven by a single model architecture. The slight performance advantage of XGBoost over the other tree-based models should be interpreted cautiously. Although XGBoost achieved the most favorable overall performance in the internal validation cohort, the absolute differences among XGBoost, LightGBM, and random forest were small. Given the limited size of the validation cohort, it remains uncertain whether this difference would translate into clinically meaningful improvement in independent populations. Therefore, the selection of XGBoost should be regarded as based on its overall performance within this dataset rather than as evidence of definitive clinical superiority over other tree-based algorithms.

Calibration and decision curve analysis are critical for evaluating whether a prediction model can be used in practice. A model with a high AUC may still be clinically unreliable if its predicted probabilities are systematically too high or too low. In the present study, the XGBoost model showed acceptable calibration, with a calibration curve close to the ideal reference line and a low Brier score. This suggests that the predicted probabilities were reasonably aligned with observed outcome frequencies. Decision curve analysis further indicated that the model provided a higher net benefit than treat-all or treat-none strategies across a clinically relevant threshold range. This has direct practical meaning. Rehabilitation resources are often limited, and clinicians must decide which patients need intensified rehabilitation, closer functional reassessment, or multidisciplinary management. A model that improves net benefit within realistic threshold probabilities may help reduce both under-treatment of high-risk patients and unnecessary escalation of care in low-risk patients (25–27).

Interpretability is another major requirement for the use of artificial intelligence in rehabilitation medicine. A black-box model that only reports a risk probability is unlikely to be accepted by clinicians, therapists, patients, or caregivers. SHAP analysis allowed us to examine how each predictor contributed to the model output at both global and individual levels (18). The most influential predictors identified by SHAP were clinically credible, which supports the transparency of the model. More importantly, SHAP explanations can help clinicians understand why a specific patient is classified as high risk. For one patient, the risk may be mainly driven by severe neurological deficit and low functional independence; for another, it may be driven by older age, multiple comorbidities, chronic-stage status, and limited rehabilitation exposure. These different risk profiles may lead to different clinical actions, even if the final predicted probability is similar.

It should also be emphasized that SHAP values do not indicate causal effects. SHAP analysis explains how the trained model uses each predictor to generate individualized risk estimates, but it does not determine whether a variable directly causes the outcome. Therefore, the negative SHAP contributions observed for structured rehabilitation therapy, acupuncture therapy, or traditional Chinese medicine-based treatment should be interpreted only as associations with lower model-predicted risk within this dataset. Because treatment allocation was observational and may have been influenced by clinical severity, physician judgment, patient preference, treatment availability, and rehabilitation tolerance, these findings are susceptible to confounding by indication. Prospective studies or randomized controlled designs are required to determine whether specific rehabilitation interventions causally improve outcomes.

Risk stratification is the practical bridge between model output and rehabilitation planning. In this study, the model separated patients into low-, intermediate-, and high-risk groups with a clear gradient of observed poor rehabilitation outcomes. This finding is important because an isolated probability value may be difficult to translate into bedside decisions. In contrast, risk categories can be linked to a structured care pathway. Low-risk patients may receive standard rehabilitation and routine follow-up. Intermediate-risk patients may benefit from more frequent reassessment, adjustment of training intensity, and early identification of delayed response. High-risk patients may require multidisciplinary rehabilitation planning, closer monitoring of neurological and functional status, proactive secondary prevention, caregiver involvement, discharge preparation, and community-transition planning. Such a framework is consistent with the goal of precision rehabilitation: matching the intensity and content of care to the patient's evolving recovery profile.

Traditional Chinese medicine-related variables provided modest incremental information but did not dominate the predictive structure. This balanced result is clinically reasonable. In real-world rehabilitation settings where acupuncture or traditional Chinese medicine-based therapies are used, these variables may reflect symptom clusters, treatment selection, patient preference, and stage-related clinical features that are not fully captured by conventional scales. At the same time, the core predictors remained neurological severity, functional status, age, disease stage, and comorbidity burden. This positioning is important for international interpretation. The model is not intended to replace standard neurological assessment with a traditional medicine framework. Rather, it treats traditional Chinese medicine-related information as one component of real-world patient phenotyping. This approach preserves the international relevance of the model while allowing local rehabilitation practice to be represented more accurately.

Another implication of the present work is that prediction should be considered a repeated clinical process rather than a one-time calculation. Stroke rehabilitation is characterized by frequent changes in neurological status, mobility, endurance, mood, caregiver support, and treatment adherence. A patient who appears high risk at admission may improve rapidly after early mobilization and structured therapy, whereas another patient initially categorized as intermediate risk may deteriorate because of infection, recurrent vascular events, depression, or poor participation. Therefore, the model should ultimately be implemented as a dynamic tool that can be updated when new information becomes available. Repeated prediction at key transition points, such as admission, early reassessment, discharge planning, and community follow-up, may provide a more realistic basis for precision rehabilitation than a single baseline estimate (38–40).

The study has several strengths. First, the model was designed specifically for rehabilitation-oriented outcome prediction rather than for general stroke prognosis. Second, it incorporated patients from different stages of post-stroke care, making it more aligned with real clinical rehabilitation pathways. Third, the model used routinely available variables, which increases the feasibility of future clinical implementation. Fourth, multiple algorithms were compared, and model performance was evaluated not only by discrimination but also by calibration, clinical net benefit, and interpretability. Fifth, the final output was translated into a risk-stratified rehabilitation framework rather than being limited to statistical reporting. This strengthens the potential clinical value of the study.

Several limitations should also be considered. First, the moderate sample size represents an important limitation of this study. Although 268 patients and 104 poor rehabilitation outcomes were included, only 188 patients and 73 outcome events were available for model development, which may increase the risk of overfitting, particularly for flexible machine learning algorithms. Feature selection, five-fold cross-validation, regularization, tree-depth restriction, subsampling, and early stopping were used to reduce overfitting and optimism; however, these procedures do not establish definitive model stability. The limited number of patients and outcome events in the training cohort remains an important constraint. Therefore, the present model should be regarded as a preliminary internally validated prediction model, and larger multicenter external validation is required to confirm its stability, transportability, and clinical usefulness (36, 37). Second, the model was developed and tested using data from a single institution and was evaluated only through an internal train-validation split. Although the internal validation results were encouraging, they do not necessarily indicate that the model would perform similarly in independent populations or different rehabilitation settings. Differences in patient characteristics, rehabilitation protocols, clinical documentation, treatment availability, and follow-up patterns may affect model transportability. Therefore, the present model should not be considered ready for direct clinical implementation. External validation in larger multicenter cohorts, followed by prospective evaluation and model updating, is required before the model can be applied in routine clinical practice. Third, the definition of poor rehabilitation outcome should be considered. We used a composite endpoint based on MBI and NIHSS improvement because both measures were routinely available and reflected complementary aspects of recovery: functional independence and neurological deficit severity. However, this definition differs from commonly used stroke rehabilitation outcomes such as the modified Rankin Scale, which assesses global disability, and the Fugl-Meyer Assessment, which evaluates motor impairment and recovery. Because these measures were not consistently available in our retrospective dataset, they could not be used as primary outcomes. This may limit comparability with other studies, and future prospective validation should include standardized outcomes such as mRS, Fugl-Meyer Assessment, and domain-specific functional measures. Fourth, treatment-related variables should also be interpreted with caution. Structured rehabilitation, acupuncture, and traditional Chinese medicine-based treatment were included as observed treatment exposures to reflect real-world rehabilitation pathways. However, treatment allocation was not randomized and may have been influenced by clinical severity, physician judgment, patient preference, and treatment availability. Therefore, these variables are susceptible to confounding by indication and should not be interpreted as evidence of treatment effectiveness.

Fifth, the study relied on routinely collected clinical variables. Important predictors such as infarct location, lesion volume, corticospinal tract integrity, cognitive status, depression, frailty, socioeconomic status, rehabilitation dose, and wearable sensor-derived activity data were not fully available. Incorporating neuroimaging, digital biomarkers, patient-reported outcomes, and detailed rehabilitation intensity may further improve performance and allow more precise recovery-trajectory modeling (16, 28, 29). Sixth, SHAP analysis improves transparency but does not establish biological mechanisms. SHAP values explain how a trained model uses predictors to generate risk estimates; they do not prove that modifying a predictor will change the outcome. Finally, the model has not yet been prospectively tested in a clinical workflow. Future research should include multicenter external validation, prospective impact analysis, model updating, and evaluation of whether model-guided rehabilitation planning improves functional outcomes, resource allocation, clinician efficiency, and patient satisfaction (25, 26, 30).

In conclusion, this study developed and internally validated an artificial intelligence-driven precision rehabilitation model for predicting poor rehabilitation outcome in patients with ischemic stroke across the continuum of care. Among the evaluated models, XGBoost showed favorable discrimination, acceptable calibration, and potential clinical utility in the internal validation cohort. Explainable artificial intelligence identified baseline functional independence, neurological severity, age, structured rehabilitation exposure, rehabilitation stage, and comorbidity burden as key contributors to individualized risk estimation. By translating predicted probabilities into clinically interpretable risk categories, the model may provide a preliminary framework for stage-sensitive rehabilitation risk stratification and planning. However, because the model was developed and tested using a single-institution cohort with internal validation only, external multicenter validation, prospective evaluation, and model updating are required before it can be recommended for routine clinical application.

## Data Availability

The raw data supporting the conclusions of this article will be made available by the authors, without undue reservation.

## References

[1] GBD 2021 Stroke Risk Factor Collaborators. Global, regional, and national burden of stroke and its risk factors, 1990–2021: a systematic analysis for the Global Burden of Disease Study 2021. Lancet Neurol. (2024) 23(10):973–1003. 10.1016/S1474-4422(24)00369-7 39304265 PMC12254192

[2] Feigin VL Stark BA Johnson CO Roth GA Bisignano C Abady GG. Global, regional, and national burden of stroke and its risk factors, 1990–2019: a systematic analysis for the Global Burden of Disease Study 2019. Lancet Neurol. (2021) 20(10):795–820. 10.1016/S1474-4422(21)00252-0 34487721 PMC8443449

[3] Powers WJ Rabinstein AA Ackerson T Adeoye OM Bambakidis NC Becker K. Guidelines for the early management of patients with acute ischemic stroke: 2019 update to the 2018 guidelines for the early management of acute ischemic stroke. Stroke. (2019) 50(12):e344–418. 10.1161/STR.0000000000000211 31662037

[4] Winstein CJ Stein J Arena R Bates B Cherney LR Cramer SC. Guidelines for adult stroke rehabilitation and recovery: a guideline for healthcare professionals from the American Heart Association/American Stroke Association. Stroke. (2016) 47(6):e98–e169. 10.1161/STR.0000000000000098 27145936

[5] Stinear CM Lang CE Zeiler S Byblow WD. Advances and challenges in stroke rehabilitation. Lancet Neurol. (2020) 19(4):348–60. 10.1016/S1474-4422(19)30415-6 32004440

[6] Bernhardt J Hayward KS Dancause N Lannin NA Ward NS Nudo RJ. A stroke recovery trial development framework: consensus-based core recommendations from the second stroke recovery and rehabilitation roundtable. Int J Stroke. (2019) 14(8):792–802. 10.1177/1747493019879657 31658893

[7] Langhorne P Bernhardt J Kwakkel G. Stroke rehabilitation. Lancet. (2011) 377(9778):1693–702. 10.1016/S0140-6736(11)60325-5 21571152

[8] Li X He Y Wang D Rezaei MJ. Stroke rehabilitation: from diagnosis to therapy. Front Neurol. (2024) 15:1402729. 10.3389/fneur.2024.1402729 39193145 PMC11347453

[9] AVERT Trial Collaboration Group. Efficacy and safety of very early mobilisation within 24h of stroke onset (AVERT): a randomised controlled trial. Lancet. (2015) 386(9988):46–55. 10.1016/S0140-6736(15)60690-0 25892679

[10] Pollock A Baer G Campbell P Choo PL Forster A Morris J. Physical rehabilitation approaches for the recovery of function and mobility following stroke. Cochrane Database Syst Rev. (2014) 2014(4):CD001920. 10.1002/14651858.CD001920.pub3 24756870 PMC6465059

[11] Kwakkel G Kollen BJ. Predicting activities after stroke: what is clinically relevant? Int J Stroke. (2013) 8(1):25–32. 10.1111/j.1747-4949.2012.00967.x 23280266

[12] Rost NS Bottle A Lee J-M Randall M Middleton S Shaw L. Stroke severity is a crucial predictor of outcome: an international prospective validation study. J Am Heart Assoc. (2016) 5(1):e002433. 10.1161/JAHA.115.002433 26796252 PMC4859362

[13] Veerbeek JM Kwakkel G Van Wegen EEH Ket JCF Heymans MW. Early prediction of outcome of activities of daily living after stroke: a systematic review. Stroke. (2011) 42(5):1482–8. 10.1161/STROKEAHA.110.604090 21474812

[14] Quinn TJ Langhorne P Stott DJ. Barthel Index for stroke trials: development, properties, and application. Stroke. (2011) 42(4):1146–51. 10.1161/STROKEAHA.110.598540 21372310

[15] Boyd LA Hayward KS Ward NS Stinear CM Rosso C Fisher RJ. Biomarkers of stroke recovery: consensus-based core recommendations from the stroke recovery and rehabilitation roundtable. Int J Stroke. (2017) 12(5):480–93. 10.1177/1747493017714176 28697711 PMC6791523

[16] Wang W Kiik M Peek N. A systematic review of machine learning models for predicting outcomes after stroke. J Stroke Cerebrovasc Dis. (2020) 29(4):104700. 10.1016/j.jstrokecerebrovasdis.2020.104700 32093987 PMC7170755

[17] Campagnini S Arienti C Patrini M Liuzzi P Mannini A Carrozza MC. Machine learning methods for functional recovery prediction and prognosis in post-stroke rehabilitation: a systematic review. J Neuroeng Rehabil. (2022) 19(1):54. 10.1186/s12984-022-01032-4 35659246 PMC9166382

[18] Zu W Huang X Xu T Du L Wang Y Wang L. Machine learning in predicting outcomes for stroke patients following rehabilitation treatment: a systematic review. PLoS One. (2023) 18(6):e0287308. 10.1371/journal.pone.0287308 37379289 PMC10306189

[19] Harari Y O'Brien MK Lieber RL Jayaraman A. Inpatient stroke rehabilitation: prediction of clinical outcomes using a machine-learning approach. J Neuroeng Rehabil. (2020) 17(1):71. 10.1186/s12984-020-00704-3 32522242 PMC7288489

[20] White A Saranti M d'Avila Garcez A Hope TMH Price CJ Bowman H. Predicting recovery following stroke: deep learning, multimodal data and feature selection using explainable AI. Neuroimage Clin. (2024) 43:103638. 10.1016/j.nicl.2024.103638 39002223 PMC11299565

[21] Campagnini S Sodero A Baccini M Hakiki B Grippo A Macchi C. Prediction of the functional outcome of intensive inpatient rehabilitation after stroke using machine learning methods. Sci Rep. (2025) 15(1):16083. 10.1038/s41598-025-00781-1 40341247 PMC12062331

[22] Zhu T Zhou Y Dai A Li S Zhou L Zhang X. Efficacy of acupuncture and rehabilitation therapy on brain function activation area and neurological function in ischemic stroke: a systematic review and meta-analysis. PLoS One. (2024) 19(3):e0298547. 10.1371/journal.pone.0298547 38394111 PMC10889652

[23] Tseng C-Y Hsu P-S Lee C-T Huang H-F Lan C-C Hsieh T-H. Acupuncture and traditional Chinese herbal medicine integrated with conventional rehabilitation for post-stroke functional recovery: a retrospective cohort study. Front Neurosci. (2022) 16:851333. 10.3389/fnins.2022.851333 35368268 PMC8966540

[24] Chavez LM Huang SS MacDonald I Lin JG Lee YC Chen YH. Mechanisms of acupuncture therapy in ischemic stroke rehabilitation: a literature review of basic studies. Int J Mol Sci. (2017) 18(11):2270. 10.3390/ijms18112270 29143805 PMC5713240

[25] Ke G Meng Q Finley T Wang T Chen W Ma W. LightGBM: a highly efficient gradient boosting decision tree. Adv Neural Inf Process Syst. (2017) 30:3146–54.

[26] Lundberg SM Lee SI. A unified approach to interpreting model predictions. Adv Neural Inf Process Syst. (2017) 30:4765–74.

[27] Collins GS Moons KGM Dhiman P Riley RD Beam AL Van Calster B. TRIPOD+AI statement: updated guidance for reporting clinical prediction models that use regression or machine learning methods. Br Med J. (2024) 385:e078378. 10.1136/bmj-2023-078378 PMC11019967 38626948

[28] DeLong ER DeLong DM Clarke-Pearson DL. Comparing the areas under two or more correlated receiver operating characteristic curves: a nonparametric approach. Biometrics. (1988) 44(3):837–45. 10.2307/2531595 3203132

[29] Vickers AJ Elkin EB. Decision curve analysis: a novel method for evaluating prediction models. Med Decis Making. (2006) 26(6):565–74. 10.1177/0272989X06295361 17099194 PMC2577036

[30] Van Calster B McLernon DJ Van Smeden M Wynants L Steyerberg EW. Topic group ‘evaluating diagnostic tests and prediction models’ of the STRATOS initiative. Calibration: the Achilles heel of predictive analytics. BMC Med. (2019) 17(1):230. 10.1186/s12916-019-1466-7 31842878 PMC6912996

[31] Breiman L. Random forests. Mach Learn. (2001) 45:5–32. 10.1023/A:1010933404324

[32] Chen T Guestrin C. XGBoost: a scalable tree boosting system. In: Proceedings of the 22nd ACM SIGKDD International Conference on Knowledge Discovery and Data Mining (New York: Association for Computing Machinery) (2016), 785–94. 10.1145/2939672.2939785

[33] Collins GS Reitsma JB Altman DG Moons KG. Transparent reporting of a multivariable prediction model for individual prognosis or diagnosis (TRIPOD): the TRIPOD statement. Ann Intern Med. (2015) 162(1):55–63. 10.7326/M14-0697 25560714

[34] Moons KGM Altman DG Reitsma JB Ioannidis JPA Macaskill P Steyerberg EW. Transparent reporting of a multivariable prediction model for individual prognosis or diagnosis (TRIPOD): explanation and elaboration. Ann Intern Med. (2015) 162(1):W1–W73. 10.7326/M14-0698 25560730

[35] Efthimiou O Seo M Chalkou K Debray T Egger M Salanti G. Developing clinical prediction models: a step-by-step guide. Br Med J. (2024) 386:e078276. 10.1136/bmj-2023-078276 PMC11369751 39227063

[36] Riley RD Archer L Snell KIE Ensor J Dhiman P Martin GP. Evaluation of clinical prediction models (part 2): how to undertake an external validation study. Br Med J. (2024) 384:e074820. 10.1136/bmj-2023-074820 PMC10788734 38224968

[37] Van Calster B Collins GS Vickers AJ. Performance evaluation of predictive AI models to support medical decisions: overview and guidance. BMJ Med. (2025) 4(1):e001328. 10.1136/bmjmed-2024-001328 41391983

[38] Ye D Luo H Winstein C Schweighofer N. Towards AI-based precision rehabilitation via contextual model-based reinforcement learning. J Neuroeng Rehabil. (2025) 22(1):263. 10.1186/s12984-025-01771-0 41419889 PMC12717728

[39] Liew S-L Cotton RJ Burdet E Bermúdez i Badia S Celnik P Cole JH. Collaborative AI for precision neurorehabilitation: a roadmap. J Neuroeng Rehabil. (2025) 22(1):269. 10.1186/s12984-025-01810-w 41310676 PMC12751918

[40] Saelmans A Wynants L Verstraete A. Implementation and updating of clinical prediction models. J Clin Epidemiol. (2025) 184:111734. 10.1016/j.jclinepi.2025.111734 39993583

